# Supplementary material for: Endocannabinoid and Nitric Oxide-Dependent IGF-I-Mediated Synaptic Plasticity at Mice Barrel Cortex
Source: Cells. 2022 May 14;11(10):1641. doi: 10.3390/cells11101641 (PMC9140009; doi:10.3390/cells11101641)
Supplement: Supplementary file 1 [file cells-11-01641-s001.zip › cells-1600139-supplementary.pdf]

cells

MDPI

Article

Endocannabinoid and Nitric Oxide-Dependent IGF-I-Mediated Synaptic Plasticity at Mice Barrel Cortex

José Antonio Noriega-Prieto [1,2], Laura Eva Maglio [1], Sara Ibáñez-Santana [1] and David Fernández de Sevilla [1,*]

[1] Departamento de Anatomía, Histología y Neurociencia, Facultad de Medicina, Universidad Autónoma de Madrid, 28029 Madrid, Spain; norie015@umn.edu (J.A.N.-P.); lauemaglio@gmail.com (L.E.M.); sara.ibannezs@estudiante.uam.es (S.I.-S.)

[2] Department of Neuroscience, University of Minnesota, Minneapolis, NM 55455, USA

* Correspondence: david.fernandezdesevilla@uam.es

Supplementary Materials:

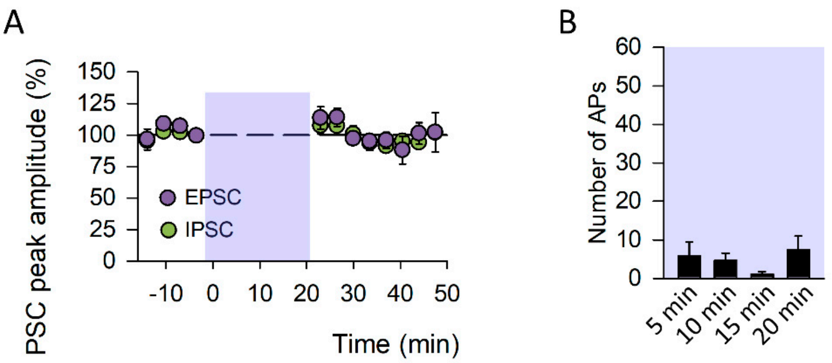

Figure S1. The protocol of stimulation does not induce plasticity. **A**) Plot showing a long-lasting stable recording of EPSCs (blue circles; n=5, *p*=0.83) and IPSCs (green circles; n=5, *p*=0.23) using the same protocol as in figure 1a, but in the absence of IGF-I. **B**) Number of action potentials recorded at different time points (5,10,15 and 20 min), showing the lack of effect of the protocol used (n=5, *p*=0.502).

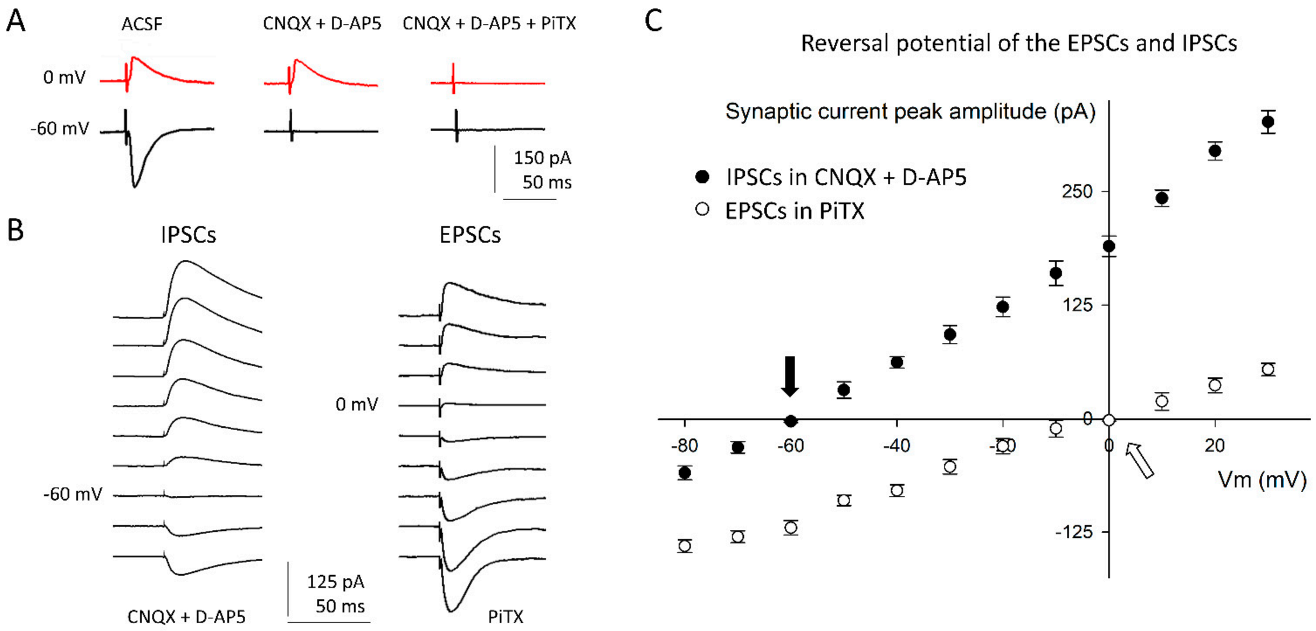

Figure S2. Reversal potential of the EPSCs and IPSCs. **A**) Representative synaptic currents recorded at 0 mV (top red recordings) and at −60 mV (bottom black recordings) in ASCF (left), in CNQX + D-AP5 (middle) and in CNQX + D-AP5 + PiTX (right). Note that the synaptic currents recorded at −60 mV are totally abolished under CNQX + D-AP5 indicating that are glutamatergic, being unaffected these recorded at 0 mV that are blocked with PiTX suggesting that are GABAergic ones. **B**) Representative IPSCs and EPSCs recorded in CNQX + D-AP5 (left) and in PiTX (right), respectively, while holding the membrane potential in steps of 10 mV. Note that −60 mV is the IPSC reversal potential whereas the EPSC reversal potential is 0 mV. C, Plot of the synaptic current peak amplitude measured at 10 mV steps from −80 mV to +30 mV in CNQX + D-AP5 (IPSCs, black circles) and in PiTX (EPSCs, white circles). Black arrow point to the IPSC reversal potential (−61.6 ± 1.7 mV, n=6) and white arrow point to the EPSC reversal potential (0 ± 2.6 mV, n=6).
